# *Chara vulgaris*-mediated selenium nanoparticles: a novel approach to antioxidant, antibacterial, anti-inflammatory, and wound healing activities

**DOI:** 10.3389/fcimb.2026.1752175

**Published:** 2026-06-09

**Authors:** Rasha Assad Assiri, Thanaa A. El-Masry, Maysa M. F. El-Nagar, Enas I. El Zahaby, Mostafa M. El-Sheekh, Abdullah A. Saber, Maisra M. El-Bouseary

**Affiliations:** 1 Department of Basic Medical Sciences, College of Medicine, Princess Nourah bint Abdulrahman University, Riyadh, Saudi Arabia; 2 Department of Pharmacology and Toxicology, Faculty of Pharmacy, Sinai University-Arish Branch, Arish, Egypt; 3 Department of Pharmacology and Toxicology, Faculty of Pharmacy, Tanta University, Tanta, Egypt; 4 Scientific Research Center and Measurements, Tanta University, Tanta, Egypt; 5 Department of Pharmaceutics, Faculty of Pharmacy, Delta University for Science and Technology, Gamasa, Egypt; 6 Botany Department, Faculty of Science, Tanta University, Tanta, Egypt; 7 Department of Biology, College of Science, Imam Mohammad Ibn Saud Islamic University (IMSIU), Riyadh, Saudi Arabia; 8 Department of Microbiology and Immunology, Faculty of Pharmacy, Tanta University, Tanta, Egypt

**Keywords:** antibacterial, anti-inflammatory, antioxidant, *Chara vulgaris* selenium nanoparticles, wound healing

## Abstract

**Background:**

*Chara vulgaris* (*C. vulgaris*), a green macroalga containing antioxidant and antibacterial compounds, shows potential in treating infections and inflammation. *Enterococcus faecalis* is an opportunistic Gram-positive pathogen responsible for skin and soft tissue infections, especially in compromised patients. Due to rising antibiotic resistance, new treatments are needed. Selenium nanoparticles (SeNPs) have enhanced antimicrobial and antioxidant properties. This study explores *C. vulgaris*-derived selenium nanoparticles (CV-SeNPs) as a therapeutic agent against *E. faecalis* infection, aiming to inhibit biofilms, reduce oxidative stress, decrease inflammation, and promote wound healing.

**Methods:**

Phenolic and flavonoid compounds estimation in the *C. vulgaris* ethanolic extract was conducted by HPLC analysis. Antioxidant activity was assessed with DPPH and ABTS radical scavenging assays. Broth dilution and crystal violet microtiter plate tests measured antibacterial and antibiofilm effects, respectively. Confocal laser scanning microscopy (CLSM) was used to observe CV-SeNPs’ effects on bacterial biofilm structure. An *in vitro* wound-healing test evaluated CV-SeNPs’ regenerative potential using normal fibroblasts. Additionally, levels of TNF-α and IL-1β were measured with ELISA kits.

**Results:**

HPLC of *C. vulgaris* ethanolic extract consists of many compounds with antioxidant, antibacterial, and anti-inflammatory activities. In DPPH and ABTS assays, CV-SeNPs showed notable antioxidant activity compared to *C. vulgaris* extract and free SeNPs. The MIC of CV-SeNPs against *E. faecalis* (*n* = 10) ranged from 128-1024 µg/ml in 70% of the tested isolates. CV-SeNPs significantly reduced bacterial biofilm formation, with the percentage of reduction of biofilm equaling 58.3% when compared to untreated cells, confirmed by CLSM observations. *In vitro* wound healing tests demonstrated that CV-SeNPs accelerated healing, with the fastest rate observed after 48 hours through cell migration into a created wound. Additionally, CV-SeNPs significantly lowered levels of TNF-α and IL-1β, indicating potential anti-inflammatory effects.

**Conclusions:**

The unique properties of CV-SeNPs suggest this compound could be an effective antioxidant, antibacterial, and anti-inflammatory agent.

## Graphical Abstract

**Figure:**
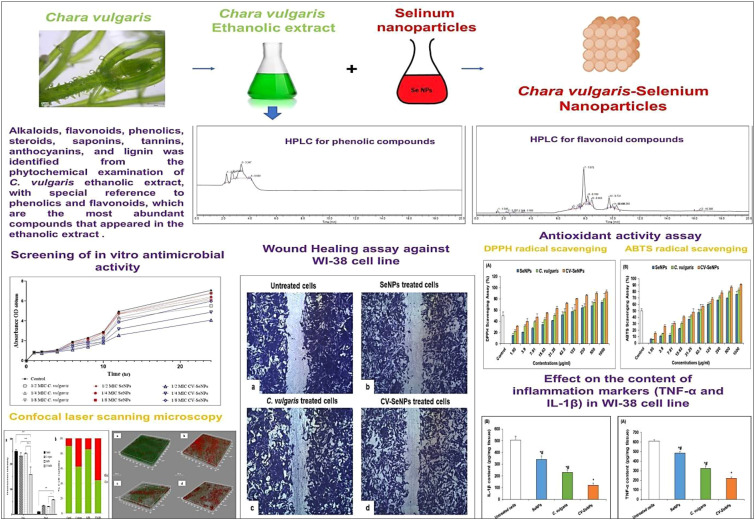

## 1 Introduction

Algae are a phylogenetically heterogeneous group of photoautotrophic microorganisms living in aquatic communities of all types. Algae range from giant seaweeds to single-cell phytoplankters. Algae exhibit a range of biological applications in medicine, such as the management of disease, wound healing, and as a source of bioactive metabolites with antimicrobial, antioxidant, and anti-inflammatory activities (Almukainzi et al., 2023; Alotaibi et al., 2024). Among them, *Chara vulgaris* (*C. vulgaris*) taxonomically belongs to the green algal group Streptophyta, and is considered to be the closest living relatives of land plants due to their structural and reproductive features. *C. vulgaris* is a submersed freshwater green alga belonging to the *Characeae* family, found in ponds, lakes, and slow-moving streams (Saber et al., 2017). *C. vulgaris* is endowed with bioactive molecules and antioxidant activity that make it an alga with varied potential medical applications (Nowak et al., 2016; Snehalatha and Rao, 2017; Shah et al., 2022). Its well-characterized antibacterial activity suggests its potential in the treatment of infections. Moreover, *C. vulgaris* is promising as a treatment for inflammatory disorders and oxidative stress-related diseases due to the anti-inflammatory and antioxidant activities of its components (Snehalatha and Rao, 2017; Maarb, 2018; Shah et al., 2022). This therapeutic potential is primarily assigned to the presence of a large amount of phenolic acids and different types of flavonoids found in *C. vulgaris*. These include ellagic acids, p-coumaric acids, and ferulic acids; and rutin, luteolin, apigenin, catechin, and quercetin. The presence of these lead compounds makes *C. vulgaris* a naturally saturating source for infection-related research work.

*Enterococcus faecalis* is a Gram-positive facultative anaerobic bacterium that, while generally an innocent gastrointestinal tract commensal, has become an important opportunistic pathogen (Rajkumari et al., 2014). Among numerous infections, skin and soft tissue infections (SSTIs) are increasingly important, particularly in patients at risk, such as diabetic patients, immunocompromised patients, or postoperative patients. SSTIs caused by *E. faecalis* can vary from cutaneous infections like cellulitis and erysipelas to more invasive infections like deep tissue abscesses, necrotizing fasciitis, and infected ulcers (Rajkumari et al., 2014; Chong et al., 2017). In chronic diabetic foot ulcers or pressure sores, *E. faecalis* typically exists as part of a polymicrobial infection, alongside other pathogens like *Staphylococcus aureus* and *Pseudomonas aeruginosa*, and facilitates delayed healing and increased inflammation (Dowd et al., 2008). The ability of *E. faecalis* to form biofilms on skin tissue, as well as on inserted medical devices like catheters and grafts, increases its persistence as well as resistance to drugs (Dowd et al., 2008). The emergence of drug-resistant strains such as vancomycin-resistant *Enterococci* (VRE) further forms a huge challenge in therapeutically treating these infections (Yadav et al., 2017). Hence, early detection, appropriate antimicrobial therapy, management of wounds, and infection control measures are prerequisites for proper *E. faecalis*-associated SSTIs management.

Selenium is a trace element that is renowned for its powerful antioxidant functions that have crucial roles to play in preserving the body’s redox balance and immune function (Almukainzi et al., 2023; Alotaibi et al., 2024). The antioxidant activity of selenium is highly important in controlling inflammation and immune response modulation, and is therefore of importance in the management of infectious diseases. Selenium has also been reported to augment immune function by motivating the activity of T-cells and macrophages and, in turn, increasing the body’s capability to resist infection (Huang et al., 2012). Selenium deficiency has also been reported to cause poor immune response and increased susceptibility to infectious organisms (Avery and Hoffmann, 2018). Selenium nanoparticles (SeNPs) showed high bioavailability, enhanced antimicrobial and antioxidant properties, and lower toxicity in comparison to bulk selenium (Almukainzi et al., 2023; Mikhailova, 2023). This is due to their high surface area, solubility, and ability to interact with microbial biofilms, and they can be used together with some natural compounds, such as *C. vulgaris*. Due to their minute size and enormous surface area, they can facilitate better interaction with microbial cells and suppress the growth of bacteria and fungi more effectively (Dowd et al., 2008; Huang et al., 2012; Rajkumari et al., 2014; Nowak et al., 2016; Chong et al., 2017; Saber et al., 2017; Snehalatha and Rao, 2017; Yadav et al., 2017; Avery and Hoffmann, 2018; Maarb, 2018; Shah et al., 2022; Almukainzi et al., 2023; Mikhailova, 2023; Alotaibi et al., 2024). A study in the recent past explored the feasibility of SeNPs against infection by exhibiting greater antimicrobial, antiviral, and anticancer properties compared to their bulk counterpart selenium (Mikhailova, 2023). Furthermore, SeNPs exhibit robust antioxidant properties, which are capable of protecting against infection-induced oxidative injury and thus hold an effective application as a weapon against infectious disease and inflammatory disease treatment (Almukainzi et al., 2023; Alotaibi et al., 2024).

Despite the scarcity of research targeting the application of *C. vulgaris* exclusively for wound healing, the use of algal organisms has long been acknowledged for having potent bioactive compounds with antioxidant and anti-inflammatory attributes, alongside the capability of tissue regeneration for the facilitation of wound healing processes (Aher et al., 2025; Hashim et al., 2025). However, there is still very limited information regarding the application of *C. vulgaris* for wound healing in living organisms through the extraction of its bioactive compounds. Additionally, recent advances in nanotechnology have improved wound healing treatments through increased antimicrobial and regenerating properties and controlled drug delivery (Agarwal et al., 2025; Aliyev et al., 2025; Banerjee et al., 2025). Different phyto-fabricated nanomaterials, such as metallic, polymeric, and composite nanoparticles, have shown promising outcomes in both preclinical and clinical trials (Mohanta et al., 2023; Sharifi-Rad et al., 2024b; Sharifi-Rad et al., 2024a). This aspect opens a new avenue for treatment in regenerative medicine, with platforms that have multiple functions, such as accelerating wound healing and improving tissue repair.

The present study proposes a novel approach for the green synthesis of selenium nanoparticles (SeNPs) in which the *C. vulgaris* is utilized as a bio-source of phenolic acids and flavonoids, which act as natural reducing and stabilizing agents during nanoparticle formation. Also, this study was designed to explore the potential of *C. vulgaris*-sourced selenium nanoparticles (CV-SeNPs) in targeted treatment of *Enterococcus faecalis* infection. Through synergistically integrating the antioxidant and antimicrobial potential of *C. vulgaris* with the state-of-the-art delivery potential of SeNPs, the methodology aims to accelerate infection control, promote biofilm disruption, and increase wound healing.

## 2 Materials and methods

### 2.1 *Chara vulgaris* collection and extract preparation

*C. vulgaris* was sampled from a very small freshwater pool at Baris Oasis, the Egyptian Western Desert, of Egypt during June 3^rd^ 2022. The ethanolic extract was prepared by adding 10 g of powder of *C. vulgaris* to 100 mL of 80% ethanol and shaking for 72 h at 25 °C as described in (Alsunbul et al., 2025). The chemical composition of *C. vulgaris* ethanolic extract (3 µL sample volume of 10 mg diluted in 1.5 mL ethanol (80%) was performed using Gas chromatography-mass spectrometry (GC-MS) analysis (Thermo Scientific’s Trace GC-ISQ mass spectrometer) as noted in (Alsunbul et al., 2025).

### 2.2 *C. vulgaris* selenium nanoparticles preparation and characterization

The ethanolic extract of *C. vulgaris* was encapsulated within selenium nanoparticles using a modified chemical reduction method; Sodium selenite (50 mg) was mixed with 10 mL of *C. vulgaris* ethanolic extract (equivalent to 100 mg) and ascorbic acid (100 mg) was employed to ensure the chemical reduction of sodium selenite into molecular selenium (ratio of 1:2 W/W), while pluronic F-127 (50 mg) was added to enhance the nanoparticle stability (Alsunbul et al., 2025). The ultrasound probe was used to decrease particle agglomeration. A standard calibration curve was prepared by serial dilution of *C. vulgaris* ethanolic extract in the range of 100-900 µg/mL to estimate the average drug content of selenium colloidal dispersion, and samples were measured using a spectrophotometric method (Shimadzu UV-VIS spectrophotometer, UV-1900I, Japan). The %Entrapment efficiency was determined in triplicate (Equation 1), the results indicated the average %EE to be 64.59 ± 4.82. The standard was scanned from 190 to 900 nm, and the spectrum showed a characteristic absorbance peak of selenium nanoparticles at λ max 268 nm (starting at 240 and ending at 300 nm) (Alsunbul et al., 2025).

(1) $$\%\text{EE}=\frac{\text{Total amount of entraped C vulgaris}}{\text{total amount of added C vulgaris }(100\text{mg})}\times 100$$

Zeta potential (-11.08 ± 1.52 mV) and polydispersity index (less than 0.5) were employed to confirm the homogeneity and stability of nanoparticles. The hydrodynamic diameter of particles was determined by dynamic light scattering, while the exact morphology was identified via TEM and SEM examination. The TEM examination illustrated homogenous spherical particles less than 100 nm in diameter. The FTIR examination confirmed the contribution of polysaccharides and phenolic contents of the ethanolic extract of *C. vulgaris* to the stability of selenium nanoparticles (Alsunbul et al., 2025).

### 2.3 *In vitro C. vulgaris* phytochemical screening

*C. vulgaris* ethanolic extract powder was used to determine the presence of different phytochemical compounds (phenolic, alkaloids, flavonoids, steroids, saponins, tannins, anthocyanins, and lignin) as noted by the Harborne method (Harborne, 1984).

### 2.4 HPLC investigation of phenolic and flavonoid compounds in the *C. vulgaris* ethanolic extract

The flavonoid and phenolic content of *C. vulgaris* ethanolic extract was analyzed using an HPLC instrument (Agilent Series 1100, Agilent, Frederick, CO, USA) by (Chang et al., 2002). Twenty-five microliters of the ethanolic extract of *C. vulgaris* was injected. A particle size of 5 µm and a column dimension of 125 mm × 4.60 mm C18 column were employed in the experiment. A two-solvent gradient for phenolic acid elution (Solvent A: methanol; Solvent B: 1:25 acetic acid and water mixture). Two solvents, (A) acetonitrile and (B) 0.2% (v/v) aqueous formic acid, were used as the mobile phase for the isocratic elution (70:30) method used to isolate flavonoids. The flow rate of the solvents was one milliliter per minute. The separation was carried out at 25 °C.

### 2.5 *In vitro* antioxidant activity analysis

#### 2.5.1 DPPH assay

In the levels of *C. vulgaris*, SeNPs, and CV-SeNPs (1.95, 3.9, 7.81, 15.63, 31.25, 62.5, 125, 250, 500, and 1000 µg/mL), a DPPH free radical scavenging experiment was carried out according to the procedure as given by (González-Palma et al., 2016). The scavenging effect percentage of DPPH was calculated by using the following formula:


Scavenging of DPPH(%)=A0−A1/A0×100

Where A0 represents the control response absorbance, and A1 represents the absorbance of a test or reference sample.

Ascorbic acid was used as the control substance. IC50 was determined from a graph of sample concentration needed to scavenge 50% of the DPPH free radicals (*n* = 3).

#### 2.5.2 ABTS assay

*C. vulgaris*, SeNPs, and CV-SeNPs were examined for ABTS radical scavenging activity at several concentrations (1.95, 3.9, 7.81, 15.63, 31.25, 62.5, 125, 250, 500, and 1000 µg/mL), with a few slight modifications, in accordance with (González-Palma et al., 2016). The absorbance at 734 nm was measured using a UV-VIS Milton Roy UV/visible spectrophotometer after an incubation period of six minutes. The antioxidant activities were calculated by applying the following formula:


ABTS scavenging(%)=(A control−A sample)/A control×100

A control = Control absorption.

A sample = Sample absorbance after six min.

Gallic acid was used as the control substance. IC50 was determined from a graph of sample concentration needed to scavenge 50% of the ABTS free radicals (*n* = 3).

### 2.6 *In vitro* antibacterial activity

#### 2.6.1 Microorganisms

In the current study, we employed bacterial reference strains, including *Enterococcus faecalis* (ATCC 29212), *Staphylococcus aureus* (ATCC 25923), *Escherichia coli* (ATCC 25922), *Klebsiella pneumoniae* (ATCC 700603), *Proteus mirabilis* (ATCC 35659), and *Pseudomonas aeruginosa* (ATCC 27853). Furthermore, we included ten clinical isolates of *E. faecalis* from the culture collection of the Department of Microbiology and Immunology, Faculty of Pharmacy, Tanta University.

#### 2.6.2 Agar disc diffusion technique

This research used the bacterial reference strains to examine the antibacterial activity of free *C. vulgaris*, SeNPs, and CV-SeNPs using the agar disc diffusion (Kirby-Bauer) test (Alotaibi et al., 2023; El-Bouseary et al., 2025). We prepared Mueller–Hinton agar plates for each strain and placed four discs on them to investigate the effect of tested compounds at 1000 µg/mL. Gentamicin was used as a reference antibiotic to test the antibacterial activity of various plant-derived and natural extracts.

#### 2.6.3 Broth microdilution assay

The broth microdilution assay was performed in order to determine the minimum inhibitory concentrations (MICs) of free *C. vulgaris*, SeNPs, and CV-SeNPs against *E. faecalis* clinical isolates using 96-well microtiter plates (El-Bouseary et al., 2018).

#### 2.6.4 Growth curves of *E. faecalis* clinical isolate

A selected clinical isolate of *E. faecalis* was grown in LB broth at 37 °C in the presence or absence of ½, ¼, and ⅛ MIC concentrations of *C. vulgaris*, SeNPs, or CV-SeNPs. The optical density (OD) of both treated and control cultures was adjusted to 0.3, and absorbance readings at 600 nm were recorded at various points throughout time (1, 2, 4, 6, 8, 10, 12, and 24 h) from each culture (Sivasamy et al., 2020; Omar et al., 2024).

#### 2.6.5 *In vitro* anti-biofilm activity

The selected *E. faecalis* clinical isolate’s biofilm formation was assessed to analyse the influence of *C. vulgaris*, SeNPs, and CV-SeNPs. This evaluation used the crystal violet (CV) microtiter plate assay (Nowak et al., 2016). To conduct this test, we grew cultures in tryptic soy broth (TSB) with 1% glucose added. We kept these cultures at 37 °C for 24 h in 96-well microtiter plates. The cultures were exposed to different conditions: some had no additives, while others contained sub-MIC levels (½, ¼, and ⅛ MIC) of *C. vulgaris*, SeNPs, or CV-SeNPs. The fixed biofilms were treated with absolute methanol for 20 min, dried by air, and stained with 200 µL of 0.1% CV for 15 min. Water was utilized to wash the plates to remove excess stain, and the plates were dried. To dissolve the bound CV, we added 200 µl of 33% (v/v) glacial acetic acid. The absorbance was measured at 570 nm in a Sunrise™ microplate reader (TECAN, Switzerland) (Rawat et al., 2020).

#### 2.6.6 Confocal laser scanning microscopy

The impact of *C. vulgaris*, SeNPs, and CV-SeNPs on bacterial biofilm formation was evaluated using confocal laser scanning microscopy (CLSM). We placed sterile glass slides in 6-well flat-bottom plates with tryptic soy broth (TSB). We added a selected *E. faecalis* clinical isolate to the broth. The broth remained untreated or received sub-MIC amounts of *C. vulgaris*, SeNPs, or CV-SeNPs. 18 h were spent incubating the plates at 37 °C. The slides underwent two PBS washes before being stained with 5 µL of each of propidium iodide (PI) and acridine orange (AO). The AO staining caused viable cells to glow green, which remained visible up to 15 min in darkness, while PI staining made dead cells fluorescent red. Finally, CLSM (DMi8, Leica Microsystems USA) was used to analyse biofilm thickness and structure (Rawat et al., 2020; Sivasamy et al., 2020). ImageJ software (version 1.49) with the COMSTAT2 plug-in was used to carry out the quantitative analysis of biofilm thickness, as well as live and dead cell distribution of the samples. A minimum of three different samples per group was analyzed to check for reproducibility.

### 2.7 Wound healing and anti-inflammatory activities

#### 2.7.1 Cell line

Human Normal lung Fibroblast (WI-38) was acquired from Nawah-Scientific Research Center.

(Almakattam Mall, Cairo, Egypt). Dulbecco’s modified Eagle’s medium/Nutrient Mixture F-12 (DMEM/F-12) (Lonza Group Ltd., Switzerland) was supplemented with 10% fetal bovine serum and 1% Penicillin-Streptomycin-Amphotericin B and was used for culturing the cells. The cells were kept in a 5% carbon dioxide incubator at 37 °C for 24 h.

#### 2.7.2 Cell viability and MTT assay

Cells were cultured on Corning 96-well tissue culture plates at 37 °C with 5% carbon dioxide in a humid atmosphere. The medium cell density was 5×10^4^ cells/well. *C. vulgaris*, SeNPs, and CV-SeNPs were added to the cells after 24 h of exponential phase growth, then. Then, 10 µL of the 12 mM stock solution of MTT (Vybrant^®^ MTT Cell Proliferation Assay Kit) was added to each well. Then, after mixing the mixture well using the pipette, 50 µL of DMSO was added to each well. It was incubated at 37 °C for 10 min. Absorbance was read at 540 nm with a Bio-Tek Instruments Inc. (Santa Clara, CA, USA) ELx 800 microplate reader (Nie et al., 2016). The optical density of treated cells (A) and control cells (B) was measured, and the inhibition rate (%) was calculated using the following equation:


Inhibition rate (%)=(B − A)/B × 100

The IC50 was also determined using the GraphPad Prism 9.0.0 software (San Diego, CA, USA) (*n* = 3).

#### 2.7.3 *In vitro* scratch assay

The cell line was divided into four groups: Control (untreated), *C. vulgaris*, SeNPs, and CV-SeNPs. Cells were seeded in a 6-well tissue culture plate at a density that, after 24 h growth, achieved 70–80% confluence. As soon as at the confluence (usually after 18–24 h), the cell layer was scraped in a straight line using a 1 mm pipette tip. The tip was kept perpendicular to the bottom of the well. A second scratch was made perpendicular to the first scratch to create a cross in each well. After scratching, the cell monolayer was washed gently to remove the cells that were scratched off, and they were substituted with fresh medium. Images were captured using an inverted microscope (CKX53, Olympus) at a magnification of 10x. After 24 and 48 h, respectively, the 6-well plate was kept in the incubator and observed under an inverted microscope until the cells had migrated to the center and closed the scratch towards the aperture (Liang et al., 2007).

#### 2.7.4 *Chara vulgaris*, SeNPs, and CV-SeNPs anti-inflammatory effect on the WI-38 cell line

The anti-inflammatory effect of *C. vulgaris*, SeNPs, and CV-SeNPs was evaluated through the estimation of TNF-α and IL-1β content in the WI-38 cell line after 24 h of incubation using ELISA kits. The ELISA kits used to determine the level of TNF-α and IL-1β were acquired from Cusabio Co., Houston, TX, USA (Cat No. CSB-E11987 and CSB-E08055, respectively). The manufacturer’s instructions were followed.

### 2.8 Statistical analysis

The data are expressed as mean values ± standard deviation (SD). Following one-way ANOVA, groups were compared using multiple comparisons with Tukey’s. Normality of the data distribution was examined using the Shapiro–Wilk test and by visual inspection of Q-Q plots and histograms. Homogeneity of variance across groups was assessed using the Brown–Forsythe test. The P-values were considered to be statistically significant if they were <0.05, <0.01, or <0.001. Statistical calculations were performed with Version 9 of GraphPad Prism (GraphPad Software Inc., La Jolla, CA, USA).

## 3 Results

### 3.1 *Chara vulgaris* ethanolic extract and its selenium nanoparticles characterization

**Figure 1:**
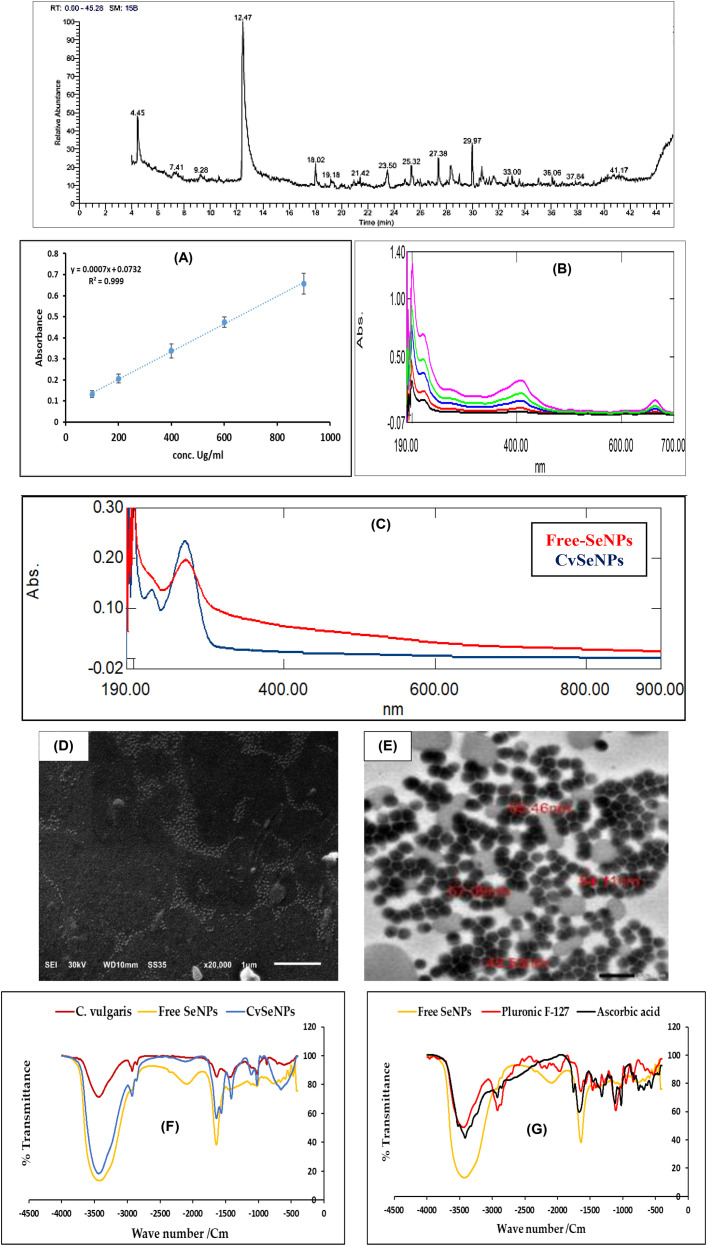
Summary of *Chara vulgaris* (*C. vulgaris*) ethanolic extract, gas chromatography-mass spectrometry and its selenium nanoparticles (CVSeNPs) characterization; Calibration curve of *C. vulgaris* **(A)**, UV spectrum of *C. vulgaris* **(B)**, spectrum of free SeNPs and CvSeNPs **(C)**, SEM of CvSeNPs **(D)**, TEM of CvSeNPs **(E)**, FTIR of *C. vulgaris*, Free SeNPs, and CvSeNPs **(F)**, and FTIR of Free SeNPs, Pluronic F-127, and ascorbic acid **(G)** as adopted from ref (Alsunbul et al., 2025)..

*C. vulgaris* ethanolic extract Gas chromatography-mass spectrometry and CV-SeNPs physicochemical characteristics (UV-Spectrum, drug content, particle size, zeta potential, polydispersity index (PDI), SEM, TEM, and FTIR analysis) were summarized in **Table 1** and **Figure 1** (Alsunbul et al., 2025).

**Table 1:** Summary of the physicochemical properties of *C. vulgaris* selenium nanoparticles as adopted from ref (Alsunbul et al., 2025).

Physiochemical property	Value
Particle size (nm)	167.85 ± 60.61
Zeta potential (mV)	-11.08 ± 1.52
PDI	0.4523 ± 0.07
Drug content (mg/mL)	28.78 ± 1.66

Data are expressed as mean ± SD, *n* = 3.

### 3.2 Phytochemical analysis in *C. vulgaris* ethanolic extract

The presence of alkaloids, flavonoids, phenolics, steroids, saponins, tannins, anthocyanins, and lignin was identified from the phytochemical examination of *C. vulgaris* ethanolic extract, with special reference to phenolics and flavonoids, which are the most abundant compounds that appeared in the ethanolic extract (**Table 2**).

**Table 2:** Phytochemical analysis of *C. vulgaris* ethanolic extract.

Phytochemical	Name of estimation test	*C. vulgaris* ethanolic extract
Alkaloids	Mayer’s test	+
Flavonoids	Ferric chloride test	++
Phenolic compounds	Phenols test	++
Steroids	Salkowski test	+
Saponins	Saponins test	+
Tannins	Ferric chloride test	+
Anthocyanin	Anthocyanin test	+
Lignin	Lignin’s test	+

### 3.3 Investigation of phenolic and flavonoid compounds in the *C. vulgaris* ethanolic extract by HPLC

**Figure 2:**
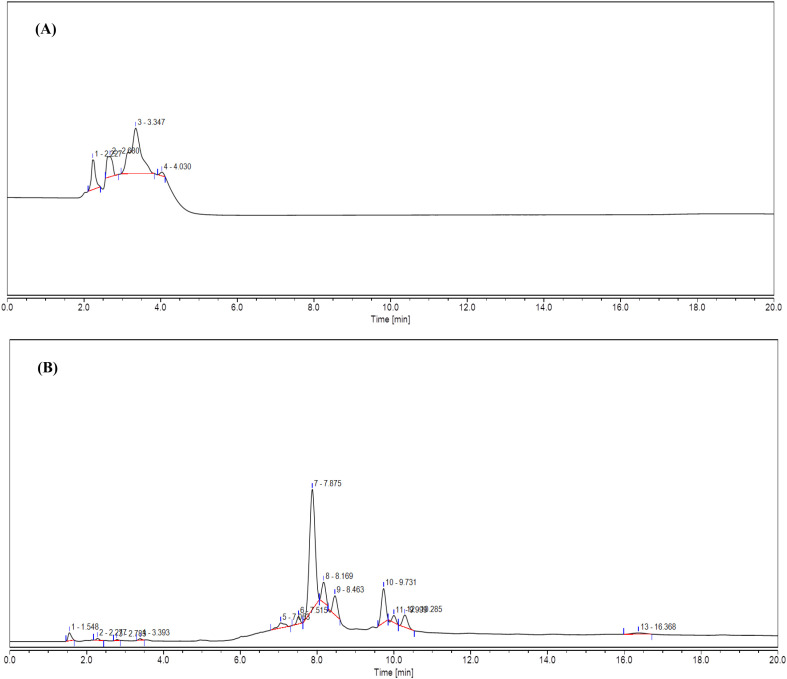
( HPLC chromatogram of phenolic **(A)** and flavonoid **(B)** compounds in the *C. vulgaris* ethanolic extract.

As shown in **Figures 2A, B**, the analysis of *C. vulgaris* ethanolic extract identified four phenolic and five flavonoid chemicals under the stated quantitative settings. The amounts of phenolic and flavonoid compounds in the ethanolic extract of *C. vulgaris* are listed in **Table 3A** and **Table 3B**.

**Table 3A:** HPLC analysis of the phenolic compounds established in the *C. vulgaris* ethanolic extract.

No.	Peak name	Retention time (min)	Area mAU*min	Relative area %	Amount µg/mL
1	Ellagic acid	2.227	1.841	16.03	32.0622
2	p-coumaric	2.680	1.842	16.04	32.0714
3	Ellagic acid	3.347	7.591	66.08	132.1645
4	Ferulic acid	4.030	0.213	1.85	3.7020

**Table 3B:** HPLC analysis of the flavonoid compounds established in the *C. vulgaris* ethanolic extract.

No.	Peak name	Retention time (min)	Area mAU*min	Relative area %	Amount µg/mL
1	Rutin	2.277	0.280	0.48	0.9512
2	Luteolin	3.393	0.234	0.40	0.7964
3	Apigenin	7.053	1.714	2.92	5.8331
4	catechin	7.875	33.623	57.21	114.4172
5	quercetin	10.285	3.020	5.14	10.2756

### 3.4 *In vitro* antioxidant activity assessment

#### 3.4.1 DPPH assay

**Figure 3:**
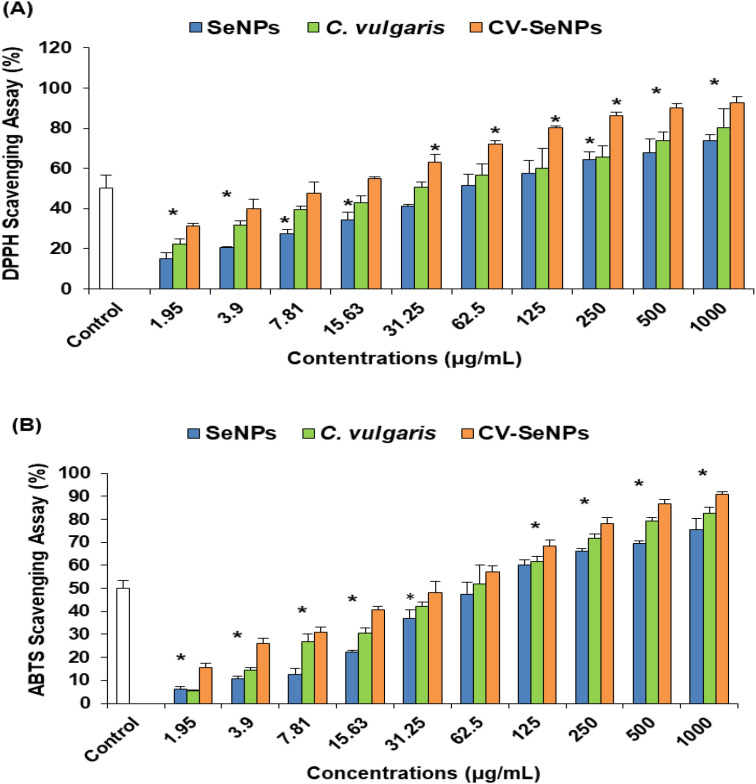
Antioxidant activity. **(A)** DPPH (Control: ascorbic acid) and **(B)** ABTS (Control: Gallic acid) SeNPs, *C. vulgaris*, and CV-SeNPs. * means significant versus the control group. Each group differed significantly from the others at *p* < 0.05.

For the evaluation of antioxidant activity, the DPPH radical scavenging activity was measured (**Figure 3A**). SeNPs, *C. vulgaris*, and CV-SeNPs treatments showed concentration-dependent DPPH radical scavenging activity (**Figure 3A**). CV-SeNPs were found to have greater activity compared to SeNPs and *C. vulgaris*. IC50 mean scavenging concentrations for SeNPs, *C. vulgaris*, and CV-SeNPs were 60.88, 30.84, and 10.05 µg/mL, respectively, while the IC50 of ascorbic acid in the positive control was 4.36 µg/mL (**Figure 3A**).

#### 3.4.2 ABTS assay

The outcomes of the ABTS and DPPH assays were consistent with each other, and the antioxidant capacity of CV-SeNPs was more significant than that of SeNPs and *C. vulgaris* (**Figure 3B**). The IC50 values of the ABTS scavenging assay were calculated to be 65.73, 60.26, and 32.36 µg/mL for SeNPs, *C. vulgaris*, and CV-SeNPs, respectively, and the IC_50_ of the positive control gallic acid was 3.71 µg/mL (**Figure 3B**).

### 3.5 *In vitro* antibacterial activity

CV-SeNPs and *C. vulgaris* displayed antibacterial activity against the *Enterococcus faecalis and Staphylococcus aureus* reference strains by the agar-well diffusion method (**Table 4**).

**Table 4:** Inhibition zone diameters (IZDs) of the tested compounds against reference bacterial strains.

Bacterial reference strain	IZD (mm)			
	*C. vulgaris*	SeNPs	CV-SeNPs	Gentamicin
*E. faecalis* (ATCC 29212)	18 ± 0.6	16 ± 0.6	20 ± 0.6^ab^	30 ± 0.6^abc^
*S. aureus* (ATCC 25923)	16 ± 0.6	11 ± 0.6^a^	16 ± 0.6^b^	28 ± 0.6^abc^
*E. coli* (ATCC 25922)	11 ± 0.6	10 ± 0.0	11 ± 0.6	26 ± 1^abc^
*K. pneumoniae* (ATCC 700603)	10 ± 0.0	10 ± 0.0	10 ± 0.0	22 ± 1^abc^
*P. aeruginosa* (ATCC 27853)	10 ± 0.6	10 ± 0.6	10 ± 0.0	21 ± 0.6^abc^
*P. mirabilis* (ATCC 35659)	12 ± 0.6	10 ± 0.0^a^	12 ± 0.6^b^	24 ± 0.6^abc^

Data were recorded as mean* ± *SD (*n* = 3). ^a^Significant vs. *C. vulgaris* group, ^b^Significant vs. SeNPs group, and ^c^Significant vs. CV-SeNPs group. Each group differed significantly from the others at *p* < 0.05.

The MIC values of *C. vulgaris*, SeNPs, and CV-SeNPs were evaluated against *Enterococcus faecalis* isolates (*n* = 10) using the broth microdilution assay. CV-SeNPs exhibited MIC values ranging from 128 to 1024 µg/mL in 70% of the isolates, whereas *C. vulgaris* displayed MIC values between 256 and 1024 µg/mL in all isolates (**Table 5**).

**Table 5:** MIC values of *C. vulgaris*, SeNPs, and CV-SeNPs against the tested *E. faecalis* clinical isolates (*n* = 10).

MIC (µg/mL)	Number of isolates (%)			
	*C. vulgaris* free	SeNPs	CV-SeNPs	Gentamicin
8	0 (0)	0 (0)	0 (0)	1 (10)
16	0 (0)	0 (0)	0 (0)	3 (30)
32	0 (0)	0 (0)	0 (0)	4 (40)
64	0 (0)	0 (0)	0 (0)	2 (20)
128	0 (0)	0 (0)	2 (20)	0 (0)
256	2 (20)	0 (0)	1 (10)	0 (0)
512	4 (40)	0 (0)	1 (10)	0 (0)
1024	1 (10)	0 (0)	3 (30)	0 (0)
> 1024	3 (30)	10 (100)	3 (30)	0 (0)

### 3.6 *In vitro* anti-biofilm activity

**Figure 4:**
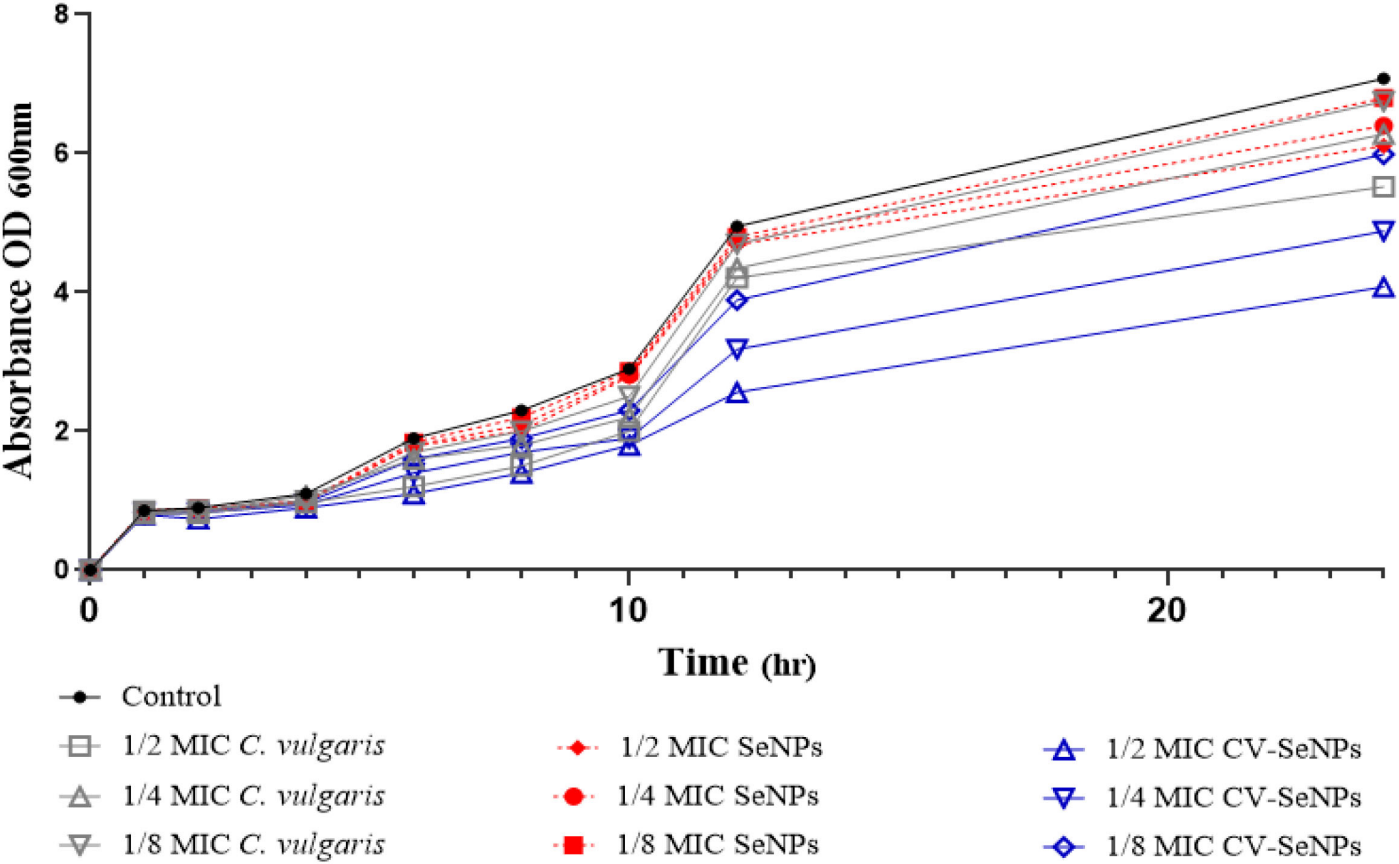
Growth curve of *E. faecalis* (E6) clinical isolate grown in the absence and presence of ½, ¼, and ⅛ MIC of tested compounds at different interval times.

The *E. faecalis* (E6) clinical isolate was selected to investigate the impact of CV-SeNPs and *C. vulgaris* on bacterial biofilm formation. We established the influence of ½, ¼, and ⅛ MIC of compounds under test on the growth curve of bacteria for determining the optimal concentrations that do not affect the growth of bacteria (**Figure 4**).

**Figure 5:**
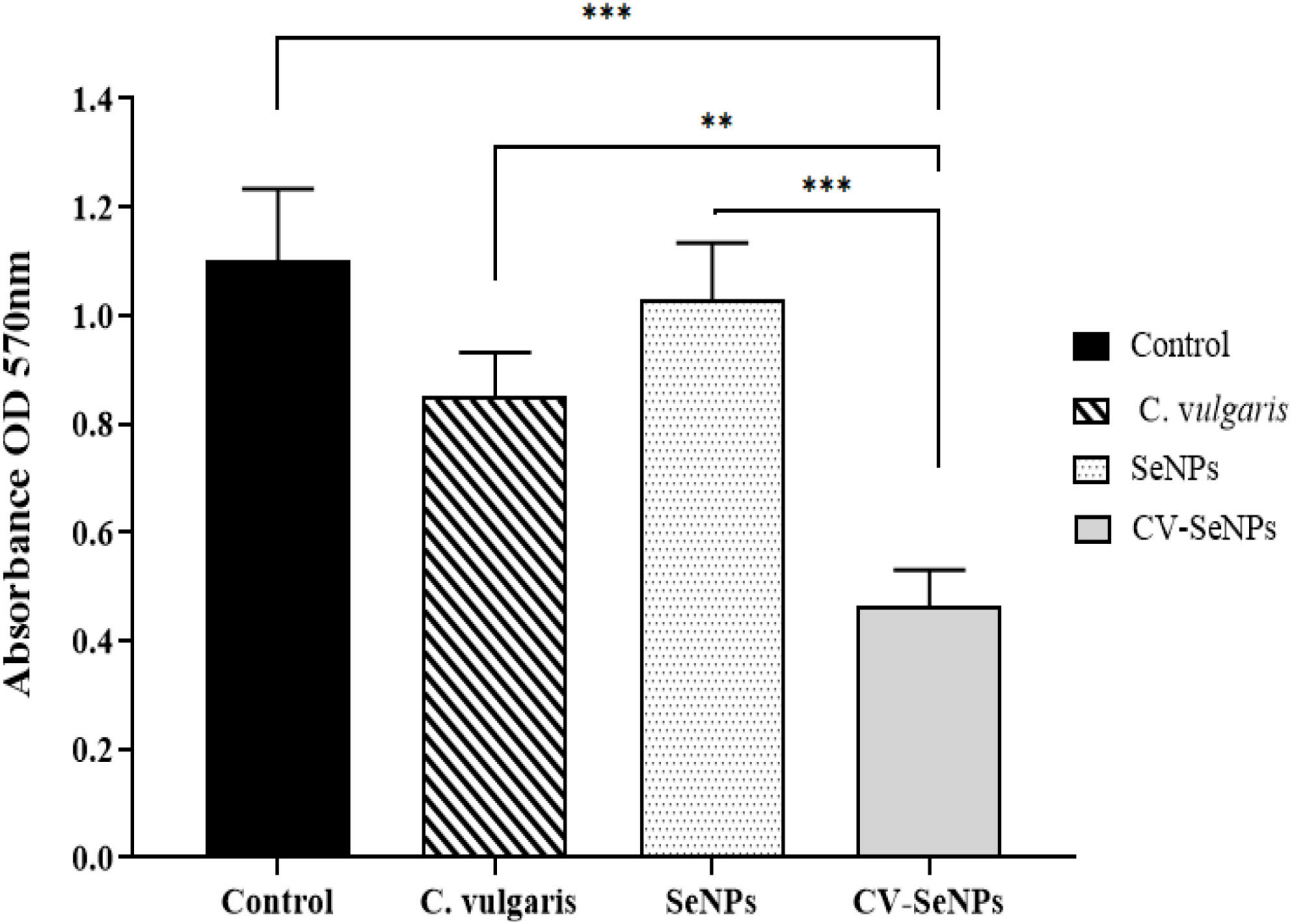
The effect of ⅛ MIC of examined compounds on biofilm formation by *E. faecalis* (E6) clinical isolate. Error bars and asterisks (*) represent standard deviations and statistically significant differences (**p < 0.01, ***p < 0.001), respectively.

The ⅛ MIC was chosen for additional studies based on the growth curve experiment results. This concentration allows for testing the antibiofilm activity of algal-based nanoparticles with minimal bacterial growth inhibition. The higher sub-MIC values (1/4 and 1/2 MIC) resulted in significant reductions that would affect the outcome of bacterial biofilm inhibition (see **Supplementary Figure S1**). The reason for choosing ⅛ MIC is supported by previous literature that showed that low doses of nanoparticles could selectively suppress bacterial biofilm formation with little to no planktonic growth inhibition (Swidan et al., 2022; Alabssawy et al., 2024). **Figure 5** represents how the compounds under study influenced bacterial biofilm formation. CV-SeNPs caused a highly significant decrease in biofilm formation (*p* < 0.001), with the percentage of reduction of biofilm equaling 58.3% when compared to untreated cells. It also resulted in a significant reduction when compared to *C. vulgaris* (*p* < 0.01), with a 45.9% reduction in biofilm formation.

### 3.7 Confocal laser scanning microscopy

**Figure 6:**
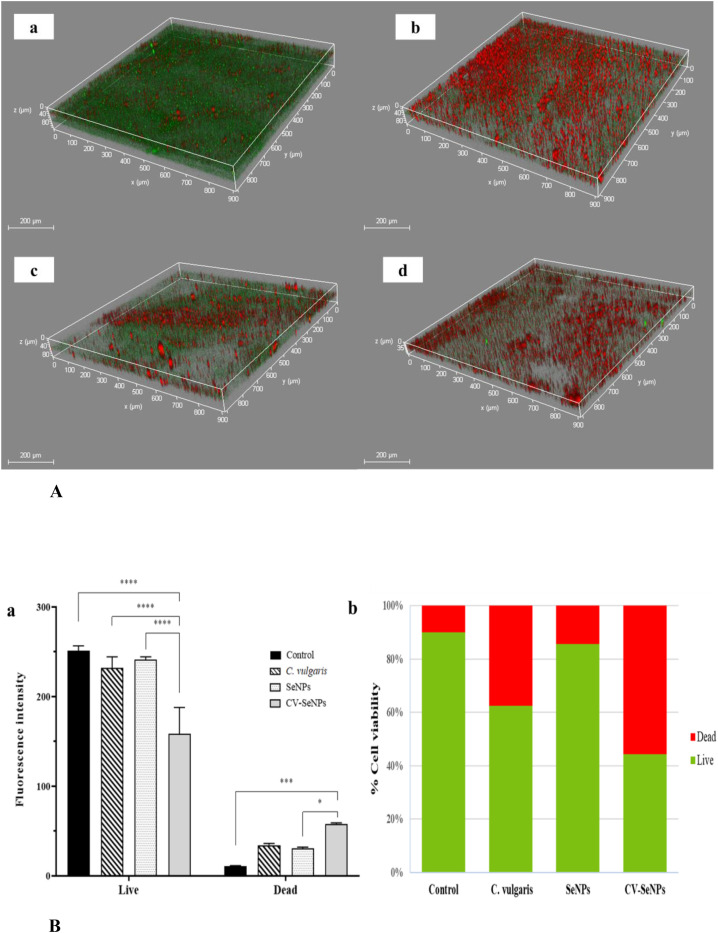
**(A)** CLSM of *E. faecalis* (E6) clinical isolate to detect biofilm thickness before and after treatment with ⅛ MIC of the tested compounds. (a) Untreated *E. faecalis* biofilm. (b) *C. vulgaris*-treated biofilm. (c) SeNPs -treated biofilm. (d) CV-SeNPs-treated biofilm. **(B)** (a) Alteration in fluorescence intensity and (b) The percentage of cell viability pre-and post-treatment with ⅛ MIC of the examined compounds. Error bars and asterisks (*) signify standard deviations and statistically significant differences (*p < 0.05, ***p < 0.001, ****p < 0.0001), respectively.

The influence of ⅛ MIC of tested compounds on *E. faecalis* (E6) biofilm formation was definite using CLSM by the double-stained technique (**Figure 6**). Viable bacterial cells were stained green by AO staining, whereas the dead bacteria were colored red by PI staining. The percent decrease of biofilm thickness after exposure to ⅛ MIC of CV-SeNPs was 56.25%. As shown in **Figure 6A**, the fluorescence intensity representing both live and dead cells revealed that CV-SeNPs displayed a highly significant reduction in live cells (*p* < 0.0001) compared to other groups and a significant increase in dead cells (*p* < 0.001) in comparison to untreated bacterial biofilm. Moreover, this observation was confirmed by calculating the percentage of cell viability (**Figure 6B**).

### 3.8 Wound healing and anti-inflammatory activities

#### 3.8.1 Cell viability MTT assay

The MTT assay displayed dose-dependent viability and inhibitory effect when using SeNPs, *C. vulgaris*, and CV-SeNPs on the WI-38 cell line incubation for 24 h. SeNPs, *C. vulgaris*, and CV-SeNPs have inhibitory percentages against the WI-38 cell line at different concentrations, with special reference to CV-SeNPs, which have the highest viability and lowest inhibition after 24 h (**Table 6**). In the MTT assay, increased cell viability corresponds to decreased inhibitory (cytotoxic) effect, and the inhibition percentage was calculated accordingly.

**Table 6:** The MTT viability test on the WI-38 cell line incubation for 24 h.

SeNPs (µg/mL)	Viability (%)	Inhibition (%)
0	100	0
16.25	80.69 ± 8.79	19.31 ± 1.2
31.25	79.38 ± 4.59	20.61 ± 1.25
62.5	78.8 ± 5.64	21.2 ± 2.09
125	77.78 ± 2.59	22.22 ± 1.08
250	67.93 ± 8.29	32.07 ± 2.21
*C. vulgaris* (µg/mL)	Viability (%)	Inhibition (%)
0	100	0
16.25	89.46 ± 1.15	10.53 ± 1.61
31.25	82.81 ± 3.7	17.19 ± 0.24
62.5	79.76 ± 2.03	20.23 ± 1.18
125	71.35 ± 9.38	28.65 ± 2.56
250	68.76 ± 12.4	31.23 ± 0.82
CVSeNPs (µg/mL)	Viability (%)	Inhibition (%)
0	100	0
16.25	92.58 ± 2.64	7.41 ± 0.26
31.25	86.61 ± 5.62	13.39 ± 0.19
62.5	83.75 ± 4.27	16.24 ± 1.40
125	81.86 ± 9.09	18.14 ± 1.52
250	70.86 ± 14.84	29.14 ± 1.81

Data are expressed as mean ± SD, *n* = 3.

#### 3.8.2 The qualitative effectiveness of SeNPs, *C. vulgaris*, and CV-SeNPs on the cell migration assay

**Figure 7:**
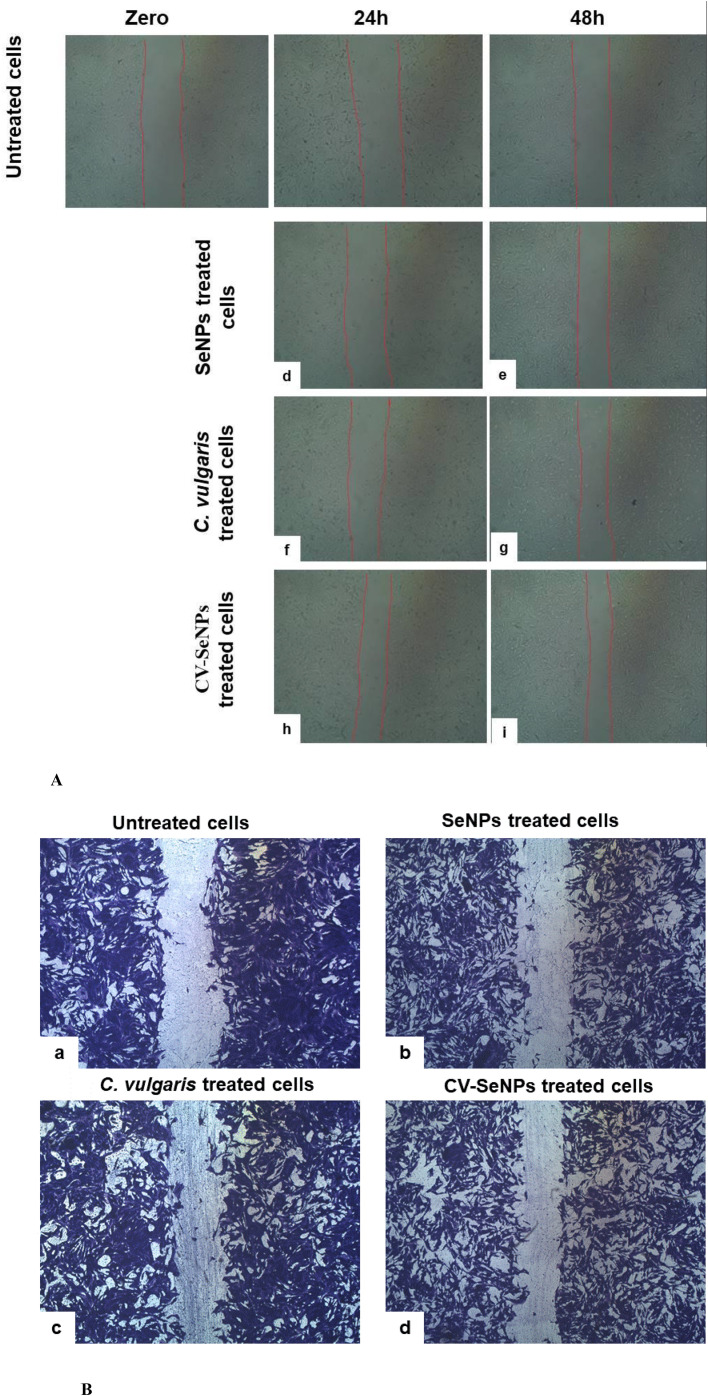
**(A)** Qualitative analysis of cell migration assay on WI-38 cells. **(B)** Qualitative analysis of cell migration assay on WI-38 cells stained with crystal violet after 48 h.

SeNPs, *C. vulgaris*, and CV-SeNPs showed an uplifted effect for the wound healing after 24 and 48 h. Furthermore, CV-SeNPs showed the quickest rate of wound healing after 48 h, based on observation of cell migration into a wound that was created on a cell monolayer (**Figures 7A, B**).

#### 3.8.3 *Chara vulgaris*, SeNPs, and CV-SeNPs effect on the inflammatory markers (TNF-α and IL-1β)

**Figure 8:**
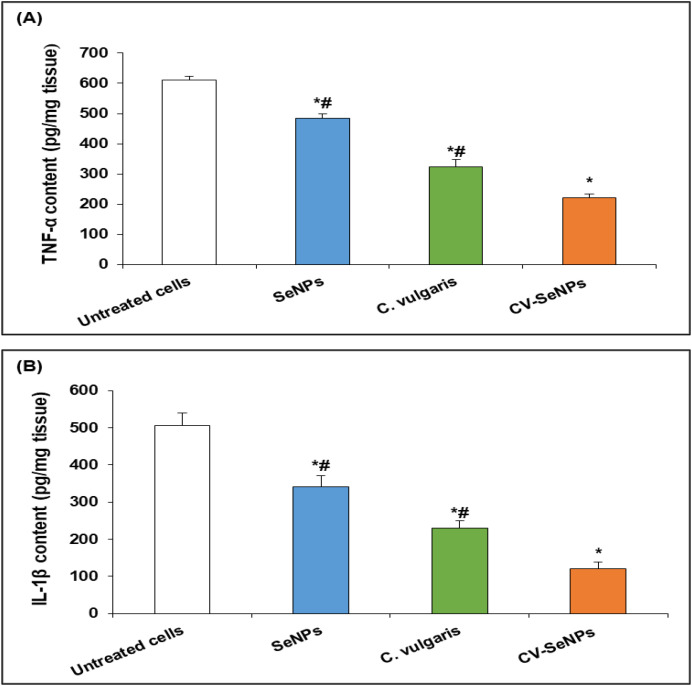
Effects of different treatments on contents of inflammatory markers TNF-α **(A)** and IL-1β **(B)**, of the wounded WI-38 cell line incubation for 24 h. Data were recorded as mean* ± *SD (*n* = 3). ^*^Significant vs. untreated cells group and ^#^significant vs. CV-SeNPs. Each group differed significantly from the others at *p* < 0.05.

SeNPs, *C. vulgaris*, and CV-SeNPs showed a reduction in the content of inflammatory markers TNF-α (20.66%, 47.07%, and 63.82%, respectively) and IL-1β (32.81%, 54.67%, and 76.21%, respectively) of the wounded WI-38 cell line incubation for 24 h in comparison to untreated cells. Furthermore, CV-SeNPs also showed the most pronounced decline in the content of TNF-α and IL-1β relative to both SeNPs and *C. vulgaris* treatment alone (**Figure 8**).

## 4 Discussion

Bioactive substances, including flavonoids, phenolics, saponins, tannins, alkaloids, and glycosides, are abundant in *chlorophyceae* algae and are responsible for their notable anti-inflammatory, antibacterial, and antioxidant properties (Liang et al., 2007; Lauritano et al., 2016). The antioxidant capacity is mostly controlled by the scavenging of free radicals by flavonoids and phenolics (Devi et al., 2011), whereas alkaloids and tannins are recognized for their antibacterial and anti-inflammatory properties, respectively (Liang et al., 2007; Matanjun et al., 2008; Lauritano et al., 2016; Mukherjee et al., 2021). The recovery of these bioactives is enhanced by the extraction process, particularly when ethanol or ethanol-water mixtures are used. In the present study, we utilized the presence of such unique bioactive ingredients as reducing and stabilizing agents to fabricate green nanoparticles to avoid the need for toxic chemicals and high energy input associated with the conventional chemical and physical methods. Previous research reported the role of algal extracts in the synthesis of copper, silver, and gold nanoparticles (Hassan et al., 2021). Additionally, there have been reports on other studies concerning the synthesis of silver, silver chloride, or silver chloride nanoparticles prepared using *Chara* (Ghusoon and Buthaina, 2024; Canales-Gutiérrez et al., 2025). These data highlight the importance of utilizing *Chara* extract in green synthesis of nanoparticles with significant biological activity. Strong free radical scavengers, these biomolecules lessen oxidative stress by encouraging the synthesis of antioxidant enzymes like catalase and superoxide dismutase (Liang et al., 2007; Lauritano et al., 2016). The purpose of this work was to investigate the potential of selenium nanoparticles derived from *C. vulgaris* (CV-SeNPs) for antioxidant, antibacterial, anti-inflammatory, and wound healing activities.

The results showed the antioxidant activity of the SeNPs, *C. vulgaris*, and CV-SeNPs estimated by DPPH and ABTS radical scavenging activity, with special reference to CV-SeNPs. This antioxidant effect could be due to free radical scavenging and induction of antioxidant enzymes, including GPx, CAT, and SOD, which lessen oxidative stress and inhibit lipid peroxidation. Our results are accompanied by Alsunbul et al. (2025), who noted strong antioxidant activity of the ethanol extract of *C. vulgaris*, as shown by DPPH radical scavenging activity, indicating its potential as a treatment for illnesses linked to oxidative stress. According to research, selenium nanoparticles (SeNPs) have various biological activities (Garala et al., 2013; Shah et al., 2022). SeNPs’ antioxidant impact is achieved via the direct scavenging of free radicals as well as the activation of GPx, catalase (CAT), and superoxide dismutase (SOD) activities. Furthermore, by lowering TBAR thiobarbituric acid, SeNPs prevent lipid peroxidation (Chen et al., 2022; Almukainzi et al., 2023; Alotaibi et al., 2024). Also, gold and silver nanoparticles are useful in medicine, but SeNPs are less expensive and may be mixed with other substances to increase their efficiency (Menon et al., 2018; Estevez et al., 2023). In agreement with our findings, Estevez et al. reported that the action of selenium in redox enzymes like GPx helps to improve the effectiveness of chemotherapy and lessen damage caused by ROS (Raturi et al., 2025).

CV-SeNPs exhibited stronger antibacterial effects than *C. vulgaris* against *E. faecalis* and *S. aureus*. This was evident from the larger inhibition zones where bacteria could not grow and the lower ranges of the minimal amounts needed to inhibit bacterial growth (128–1024 µg/mL for 70% of the samples) compared to the *C. vulgaris* extract (256–1024 µg/mL). As previously reported, this significant antibacterial activity of the biosynthesized SeNPs is due to their small size, large surface area, and natural coatings from the source organism, all of which contribute to this effect. The observed antibacterial property is associated with various mechanisms such as disturbance in the bacterial cell membranes, inhibition of bacterial metabolism, and the production of ROS, causing oxidative stress in bacterial cells (Debro and Ward, 1979; Filipović et al., 2021; Mikhailova, 2023). *C. vulgaris* has a unique impact on bacterial communities, notably affecting Gram-positive bacteria (Snehalatha and Rao, 2017). Consistent with our findings, prior studies confirmed its strong activity against *S. aureus* and *B. subtilis*, while Gram-negative bacteria and yeast exhibited resistance (Jeevitha and Malammanavar, 2025). These effects originate from bioactive molecules identified in *C. vulgaris* extracts through GC-MS and phytochemical screening tests, including alkaloids, phenols, terpenes, flavonoids, resins, saponins, and tannins (Maarb, 2018; Manikanta and Malammanavar, 2018; Hashim et al., 2025). A wide range of secondary metabolites suggests that bacteria-fighting mechanisms operate in multiple ways, such as damaging cell membranes, inhibiting enzyme activity, and inducing oxidative stress (Maarb, 2018). Additionally, SeNPs also demonstrate antibacterial activity against Gram-positive bacteria like *E. faecalis* and *S. aureus*, by mechanisms involving cell wall degradation and metabolic disruption (Ali and Neelakantan, 2022). Earlier research indicated that the antibacterial effects of *C. vulgaris* and other algal extracts are enhanced when attached to nanoparticle surfaces (Maarb, 2018; Ali and Neelakantan, 2022).

*Enterococcus faecalis* exhibited a characteristic ability to form biofilms with high resistance to antibiotics, which produce complex three-dimensional structures in their extracellular matrices (Alam et al., 2019). The present study was designed to check the antibiofilm activity of the CV-SeNPs on *E. faecalis* biofilms. Although several studies have been made on *C. vulgaris* up until now, they have been focused largely on their anti-microbial activity, without exploring their structural and viability impacts on biofilm formation of *E. faecalis* (Sans-Serramitjana et al., 2023; Kumari, 2025). Hence, our study is filling that particular gap in the literature on algal nanobiotechnology for controlling bacterial biofilm formation. In the present study, we demonstrated that CV-SeNPs showed superior antibiofilm properties compared to untreated cells, SeNPs, and *C. vulgaris* extract. This improved effect is due to the fact that SeNPs can penetrate and break down the biofilm EPS matrix with the help of algal-derived capping agents that suppress bacterial growth and attachment (Maarb, 2018; Jeevitha and Malammanavar, 2025). Moreover, SeNPs generate reactive oxygen species, leading to oxidative stress that harms embedded cells and reduces the expression of biofilm-related genes (Maarb, 2018). The combination of nanoparticle physicochemical action and phytochemical cooperation has an impact on biofilm structure and thickness, leading to improved effectiveness. Mechanistically, selenium nanoparticles (SeNPs) interfere with the extracellular polymeric substance (EPS) matrix of the biofilm, while reactive oxygen species (ROS) target the bacteria sequestered within and reduce the expression of genes related to the biofilm, resulting in a thinner and less viable biofilm (Maarb, 2018; Manikanta and Malammanavar, 2018; Jeevitha and Malammanavar, 2025). These findings are in line with previous reports that SeNPs produced through plant- and algal-mediated synthesis show a more potent antibiofilm activity (Mandal et al., 2023). These findings are confirmed with the results of confocal laser scanning microscopy (CLSM), which revealed that exposure to ⅛ MIC of CV-SeNPs caused a disruptive effect on the *E. faecalis* (E6) biofilm structure. Compared to the control, a reduced thickness of the biofilm matrix is observed. The use of double-fluorescence staining methods reveals a significant reduction in viable cell counts, a result supported by reduced green fluorescence intensity. Also, the significant increase in non-viable cell population is authenticated by increased red fluorescence. Quantitative measurements verify these findings. Taken together, these findings indicate a significant shift towards reduced viability of treated biofilms.

The impact of CV-SeNPs on wound healing was also examined *in vitro*, and based on the observation of cell migration into a wound formed on a cell monolayer, CV-SeNPs demonstrated the fastest rate of wound healing after 48 h. Additionally, CV-SeNPs showed the highest downregulation of IL-1β and TNF-α, confirming their possible anti-inflammatory action. It is well acknowledged that a wound heals in three major phases after hemostasis. During the first stage, inflammation occurs, which causes neutrophils and macrophages to gather at the lesion site.

The next step is proliferation, when granulation tissue formation, angiogenesis, re-epithelization, as well as fibroplasia (Canales-Gutiérrez et al., 2025). Finally, neovascularization and the start of neocollagenesis define the remodeling phase. It takes longer for the wound to heal in this last stage.

The two primary goals of wound-healing therapies are to shorten the healing period and prevent undesirable side effects like scarring (Canales-Gutiérrez et al., 2025).

According to reports, many reasons can make wound healing difficult, for example, sepsis caused by a secondary bacterial infection, possibly by *E. faecalis* or *S. aureus*. Moreover, by the activation of inflammatory cytokines, inflammation plays a serious role in wound healing (Okur et al., 2020). Pro-inflammatory cytokines like IL-1β and TNF-α are essential for triggering inflammation, attracting neutrophils, removing pathogens and pollutants from the injury site, and encouraging the production of metalloproteinases (MMPs). By eliminating damaged extracellular matrices (ECMs), these MMPs aid in tissue rebuilding and contribute to the healing process (Alsenani et al., 2021). On the other hand, because the cytokines and proteinases generated by continuous inflammation may intensify tissue damage, chronic wounds may result (Schilrreff and Alexiev, 2022). Additionally, inflammation causes the surrounding tissues to be badly damaged, and the injury may progress to a pathological condition that requires more extensive treatment (Mitra et al., 2025). Anti-inflammatory drugs are therefore crucial for the treatment of wounds (Alim et al., 2019). Overall, the observed increase in wound healing and cell migration may be explained by the down-regulation of pro-inflammatory cytokines (IL-1β and TNF-α), reduced oxidative stress, and enhanced tissue regeneration. Such regeneration encompasses angiogenesis, epithelialization, and fibroplasia. All these occur due to the phyto-stimulating action of *C. vulgaris*.Therefore, the development of new drugs based on natural products on a nanoscale complex has significantly improved the capabilities of existing medicines, and gives the chance for newly synthesized drugs that may have many useful therapeutic properties to be used. Consequently, the combination of *C. vulgaris* ethanolic extract, which has a variety of bioactive molecules (Alsunbul et al., 2025) and SeNPs that have also many biomedical applications due to their notable properties (Ferro et al., 2021; Shahabi et al., 2021), may enhance their biological functions as anticancer, antioxidant, anti-inflammatory, and antidiabetic agents. Besides, SeNPs can be employed as carriers to deliver drugs to specific tissues.

## 5 Conclusion

As far as we know, this is the first report of *C. vulgaris* and CV-SeNPs’ antibacterial and antibiofilm properties against *S. aureus* or *E. faecalis*. DPPH and ABTS radical scavenging assay results also corroborated our findings that suggested *C. vulgaris* and CV-SeNPs have notable antioxidant activity. They also showed remarkable anti-inflammatory activity since the downregulation of IL-1β and TNF-α. Moreover, CV-SeNPs had the highest healing rate of the wound. We suggest more investigation on this formula in the antibacterial and inflammation control area based on the special property of CV-SeNPs.

## Data Availability

The raw data supporting the conclusions of this article will be made available by the authors, without undue reservation.

## References

[B1] Agarwal G. Bhargava S. Dumoga S. (2025). Nanomaterial interventions for wound healing: Current status of preclinical and clinical studies. Wound Repair Regen. 33, e70031. doi:10.1111/wrr.70031 40322951

[B2] Aher A. A. Thitame S. N. Viddyasagar M. Wabale A. S. (2025). Bioactive algae in wound healing: natural solutions for modern medicine. J. Pharm. Bioallied Sci. 17, S20–S23. doi:10.4103/jpbs.jpbs_1639_24 40511231 PMC12156601

[B3] Alabssawy A. N. Abu-Elghait M. Azab A. M. Khalaf-Allah H. M.M. Ashry A. S. Ali A. O.M. . (2024). Hindering the biofilm of microbial pathogens and cancer cell lines development using silver nanoparticles synthesized by epidermal mucus proteins from Clarias gariepinus. BMC Bio/Technol. 24, 28. doi:10.1186/s12896-024-00852-7 38702622 PMC11069147

[B4] Alam H. Khatoon N. Raza M. Ghosh P. C. Sardar M. (2019). Synthesis and characterization of nano selenium using plant biomolecules and their potential applications. Bionanosci. 9, 96–104. doi:10.1007/s12668-018-0569-5 30311153

[B5] Ali I. A. A. Neelakantan P. (2022). Antibiofilm activity of phytochemicals against Enterococcus faecalis: A literature review. Phytother. Res. 36, 2824–2838. doi:10.1002/ptr.7488 35522168

[B6] Alim I. Caulfield J. T. Chen Y. Swarup V. Geschwind D. H. Ivanova E. . (2019). Selenium drives a transcriptional adaptive program to block ferroptosis and treat stroke. Cell. 177, 1262–1279.e25. doi:10.1016/j.cell.2019.03.032 31056284

[B7] Aliyev A. Israyilova A. Hasanova U. Gakhramanova Z. Ahmadova A. (2025). Nanotechnology in wound healing: A new frontier in regenerative medicine. Micro. 5, 60. doi:10.3390/micro5040060 30654563

[B8] Almukainzi M. El-Masry T. A. Selim H. Makhlof M. E. M. El-Bouseary M. M. (2023). New insight on the cytoprotective/antioxidant pathway Keap1/Nrf2/HO-1 modulation by Ulva intestinalis extract and its selenium nanoparticles in rats with carrageenan-induced paw edema. Mar. Drugs 21, 459. doi:10.3390/md21090459 37755072 PMC10533125

[B9] Alotaibi B. Elekhnawy E. El-Masry T. A. Saleh A. El-Bouseary M. M. Alosaimi M. E. . (2023). Green synthesized Cu-oxide nanoparticles: properties and applications for enhancing healing of wounds infected with Staphylococcus aureus. Int. J. Pharm. 645, 123415. doi:10.1016/j.ijpharm.2023.123415 37714313

[B10] Alotaibi B. S. El-Masry T. A. Selim H. El-Bouseary M. M. El-Sheekh M. M. Makhlof M. E. M. . (2024). New insights into the anticancer effects of Polycladia crinita aqueous extract and its selenium nanoformulation against the solid Ehrlich carcinoma model in mice via VEGF, Notch 1, NF-κB, cyclin D1, and caspase 3 signaling pathway. Front. Pharmacol. 15, 1345516. doi:10.3389/fphar.2024.1345516 38469406 PMC10926956

[B11] Alsenani F. Ashour A. M. Alzubaidi M. A. Azmy A. F. Hetta M. H. Abu-Baih D. H. . (2021). Wound healing metabolites from Peters’ elephant-nose fish oil: an *in vivo* investigation supported by *in vitro* and in silico studies. Mar. Drugs 19, 605. doi:10.3390/md19110605 34822477 PMC8625051

[B12] Alsunbul M. El-Masry T. A. El-Bouseary M. M. El Zahaby E. I. El-Sheekh M. M. Gaballa M. M.S. . (2025). Evaluating the anticancer efficacy of Chara vulgaris ethanolic extract and selenium nanoformulation in Ehrlich carcinoma mice: role of autophagy and apoptosis. BMC Bio/Technol. 25, 53. doi:10.1186/s12896-025-00998-y 40597910 PMC12211136

[B13] Avery J. C. Hoffmann P. R. (2018). Selenium, selenoproteins, and immunity. Nutrients 10, 1203. doi:10.3390/nu10091203 30200430 PMC6163284

[B14] Banerjee D. Vydiam K. Vangala V. Mukherjee S. (2025). Advancement of nanomaterials- and biomaterials-based technologies for wound healing and tissue regenerative applications. ACS Appl. Bio Mater. 8, 1877–1899. doi:10.1021/acsabm.5c00075. 40019109

[B15] Canales-Gutiérrez A. Mullisaca-Torres F. Peali-Maro G. (2025). Systematic review of green nanotechnology in the Andes: nanoparticle biosynthesis and applications from Chara globularis. Rev. Amb Agua 25, e3080. doi:10.4136/ambi-agua.3080

[B16] Chang C. C. Yang M. H. Wen H. M. Chern J. C. (2002). Estimation of total flavonoid content in propolis by two complementary colometric methods. J. Food. Drug Ana. 10, 178–182. doi:10.38212/2224-6614 41525198

[B17] Chen W. Cheng H. Xia W. (2022). Progress in the surface functionalization of selenium nanoparticles and their potential application in cancer therapy. Antioxid. (Basel) 11, 1965. doi:10.3390/antiox11101965 36290687 PMC9598587

[B18] Chong K. K. L. Tay W. H. Janela B. Yong A. M. H. Liew T. H. Madden L. . (2017). Enterococcus faecalis modulates immune activation and slows healing during wound infection. J. Infect. Dis. 216, 1644–1654. doi:10.1093/infdis/jix541 29045678 PMC5854026

[B19] Debro L. H. Ward H. B. (1979). Antibacterial activity of freshwater green algae. Planta Med. 36, 375–378. doi:10.1055/s-0028-1097284 493402

[B20] Devi G. K. Manivannan K. Thirumaran G. Rajathi F. A. A. Anantharaman P. (2011). *In vitro* antioxidant activities of selected seaweeds from southeast coast of India. Asian Pac. J. Trop. Med. 4, 205–211. doi:10.1016/S1995-7645(11)60070-9. 21771454

[B21] Dowd S. E. Wolcott R. D. Sun Y. McKeehan T. Smith E. Rhoads D. (2008). Polymicrobial nature of chronic diabetic foot ulcer biofilm infections determined using bacterial tag encoded FLX amplicon pyrosequencing (bTEFAP). PloS One 3, e3326. doi:10.1371/journal.pone.0003326 18833331 PMC2556099

[B22] El-Bouseary M. El-Banna T. Sonbol F. (2018). Prevalence of MRSA among Staphylococcus aureus isolates recovered from patients with otitis media. Nat. Sci. 16, 48–55. doi:10.7537/marsnsj160618.08

[B23] El-Bouseary M. M. Eliwa D. Farghali M. H. Ragab A. E. (2025). Investigating the potential antibacterial, anti-biofilm, wound healing, and anti-inflammatory activity of the extract of Aspergillus Niger endophyte isolated from cucumber leaves: *in vitro* and *in vivo* study. BMC Microbiol. 25, 420. doi:10.1186/s12866-025-04134-w 40624613 PMC12232745

[B24] Estevez H. Garcia-Calvo E. Mena M. L. Alvarez-Fernandez Garcia R. Luque-Garcia J. L. (2023). Unraveling the mechanisms of Ch-SeNP cytotoxicity against cancer cells: insights from targeted and untargeted metabolomics. Nanomaterials (Basel) 13, 2204. doi:10.3390/nano13152204 37570523 PMC10420838

[B25] Ferro C. Florindo H. F. Santos H. A. (2021). Selenium nanoparticles for biomedical applications: From development and characterization to therapeutics. Adv. Healthcare Mater. 10, 2100598. doi:10.1002/adhm.202100598 34121366

[B26] Filipović N. Ušjak D. Milenković M. T. Zheng K. Liverani L. Boccaccini A. R. . (2021). Comparative study of the antimicrobial activity of selenium nanoparticles with different surface chemistry and structure. Front. Bioeng. Biotechnol. 8, 624621. doi:10.3389/fbioe.2020.624621 33569376 PMC7869925

[B27] Garala K. Joshi P. Shah M. Ramkishan A. Patel J. (2013). Formulation and evaluation of periodontal in situ gel. Int. J. Pharm. Investig. 3, 29–41. doi:10.4103/2230-973X.108961 23799203 PMC3687234

[B28] Ghusoon A. A. A. Buthaina A. H. A. (2024). Investigation of the effect of the aqueous extract of Chara vulgaris (L.) on visceral leishmaniasis. Trop. Parasitol. 14, 84–94. doi:10.4103/tp.tp_1_24 39411680 PMC11473012

[B29] González-Palma I. Escalona-Buendía H. B. Ponce-Alquicira E. Téllez-Téllez M. Gupta V. K. Díaz-Godínez G. . (2016). Evaluation of the antioxidant activity of aqueous and methanol extracts of Pleurotus ostreatus in different growth stages. Front. Microbiol. 7, 1099. doi:10.3389/fmicb.2016.01099 27462314 PMC4940407

[B30] Harborne (1984). Phytochemical methods, Thomson Sciences. Available online at: https://link.springer.com/book/10.1007/978-94-009-5570-7 (Accessed August 15, 2025).

[B31] Hashim M. Khan F. U. Ullah A. Jamal F. Iqbal H. Ullah S. . (2025). The assessment of the Spirogyra varians and Chara vulgaris comparative and combined antioxidant potential. J. Med. Health Sci. Rev. 2. doi:10.62019/4nwqbz67

[B32] Hassan K. T. Ibraheem I. J. Hassan O. M. Obaid A. S. Ali H. H. Salih T. A. . (2021). Facile green synthesis of Ag/AgCl nanoparticles derived from Chara algae extract and evaluating their antibacterial activity and synergistic effect with antibiotics. J. Environ. Chem. Eng. 9, 105359. doi:10.1016/j.jece.2021.105359 38826717

[B33] Huang Z. Rose A. H. Hoffmann P. R. (2012). The role of selenium in inflammation and immunity: from molecular mechanisms to therapeutic opportunities. Antioxid. Redox Signal. 16, 705–743. doi:10.1089/ars.2011.4145 21955027 PMC3277928

[B34] Jeevitha M. P. Malammanavar S. G. (2025). Antimicrobial activity of bioactive compounds extracted from Chara globularis Thuill. J. Appl. Pharm. Res. 13 (5), 216–231. doi:10.69857/joapr.v13i5.1371

[B35] Kumari S. (2025). Charophyceae is promising for valuable phytochemical and antibacterial properties—a study. Int. J. Multidiscip. Res. (IJFMR) 7, 1. Available online at: https://www.ijfmr.com/papers/2025/6/61989.pdf.

[B36] Lauritano C. Andersen J. H. Hansen E. Albrigtsen M. Escalera L. Esposito F. . (2016). Bioactivity screening of microalgae for antioxidant, anti-inflammatory, anticancer, antidiabetes, and antibacterial activities. Front. Mar. Sci. 3, 68. doi:10.3389/fmars.2016.00068

[B37] Liang C. C. Park A. Y. Guan J. L. (2007). *In vitro* scratch assay: a convenient and inexpensive method for analysis of cell migration *in vitro*. Nat. Protoc. 2, 329–333. doi:10.1038/nprot.2007.30 17406593

[B38] Maarb S. (2018). Al-maoula Ahmed S. Dwaish. Anti-Dermatophytes activity of some algal extracts isolated from Baghdad City, Iraq. Res. J. Pharm. Tech. 11, 5449–5454. doi:10.5958/0974-360X.2018.00993.9

[B39] Mandal A. K. Nayak R. Pradhan B. Behera C. Behera A. K. Parida S. . (2023). Algal-derived nanoparticles and their antibacterial potential: current evidence and future perspectives. J. Microbiol. Methods 211, 106790. doi:10.1016/j.mimet.2023.106790 37487886

[B40] Manikanta G. S. Malammanavar S. G. (2018). Phytochemistry and antimicrobial activity of Chara. J. Pharmacogn. Phytochem. 7, 2047–2050. Available online at: https://www.phytojournal.com/archives/2018/vol7issue6/PartAJ/7-6-138-571.pdf.

[B41] Matanjun P. Mohamed S. Mustapha N. M. Muhammad K. Ming C. H. (2008). Antioxidant activities and phenolic content of eight species of seaweed from North Borneo. J. Appl. Phycol. 20, 367–373. doi:10.1007/s10811-007-9264-6 30311153

[B42] Menon S. Ks S. D. Santhiya R. Rajeshkumar S. Kumar V. S. (2018). Selenium nanoparticles: a potent chemotherapeutic agent and an elucidation of its mechanism. Colloids Surf. B. Biointerfaces 170, 280–292. doi:10.1016/j.colsurfb.2018.06.006 29936381

[B43] Mikhailova E. O. (2023). Selenium nanoparticles: green synthesis and biomedical application. Molecules 28, 8125. doi:10.3390/molecules28248125 38138613 PMC10745377

[B44] Mitra A. Shahid A. Kumari S. Mukherjee T. Pramanick S. Mohanty S. . (2025). Optimizing wound healing: insights from phytochemicals and advanced therapies. Inflammopharmacology 33, 4009–4025. doi:10.1007/s10787-025-01806-x 40540111

[B45] Mohanta Y. K. Mishra A. K. Panda J. Chakrabartty I. Sarma B. Panda S. K. . (2023). Promising applications of phyto-fabricated silver nanoparticles: Recent trends in biomedicine. Biochem. Biophys. Res. Commun. 688, 149126. doi:10.1016/j.bbrc.2023.149126 37951153

[B46] Mukherjee A. Sarkar D. Sasmal S. (2021). A review of green synthesis of metal nanoparticles using algae. Front. Microbiol. 12, 693899. doi: 10.3389/fmicb.2021.693899 34512571 PMC8427820

[B47] Nie T. Wu H. Wong K. H. Chen T. (2016). Facile synthesis of highly uniform selenium nanoparticles using glucose as the reductant and surface decorator to induce cancer cell apoptosis. J. Mater. Chem. B 4, 2351–2358. doi:10.1039/c5tb02710a 32263230

[B48] Nowak P. Schubert H. Schaible R. (2016). Molecular evaluation of the validity of the morphological characters of three Swedish Chara sections: Chara, Grovesia, and Desvauxia (Charales, Charophyceae). Aquat. Bot. 134, 113–119. doi:10.1016/j.aquabot.2016.08.001 38826717

[B49] Okur M. E. Karantas I. D. Senyigit Z. Üstündag Okur N. Siafaka P. I. (2020). Recent trends on wound management: new therapeutic choices based on polymeric carriers. Asian J. Pharm. Sci. 15, 661–684. doi:10.1016/j.ajps.2019.11.008 33363624 PMC7750807

[B50] Omar A. El-Banna T. E. Sonbol F. I. El-Bouseary M. M. (2024). Potential antivirulence and antibiofilm activities of sub-MIC of oxacillin against MDR S. aureus isolates: an *in vitro* and *in vivo* study. BMC Microbiol. 24, 1–18. doi:10.1186/s12866-024-03429-8 39123138 PMC11312681

[B51] Rajkumari N. Mathur P. Misra M. C. (2014). Soft tissue and wound infections due to Enterococcus spp. among hospitalized trauma patients in a developing country. J. Glob. Infect. Dis. 6, 189–193. doi:10.4103/0974-777X.145253 25538459 PMC4265836

[B52] Raturi P. Ahmad N. Rawat N. Singhvi N. (2025). Synthesis and biomedical-based applications of selenium nanoparticles: A comprehensive review. Indian J. Microbiol. 65, 204–215. doi:10.1007/s12088-024-01302-w 40371022 PMC12069214

[B53] Rawat L. Hegde H. Hoti S. L. Nayak V. (2020). Piperlongumine induces ROS-mediated cell death and synergizes paclitaxel in human intestinal cancer cells. Biomed. Pharmacother. 128, 110243. doi:10.1016/j.biopha.2020.110243 32470748

[B54] Saber A. A. Ballot A. Schneider S. C. Cantonati M. (2017). Morphological and molecular features of a Chara vulgaris population from desert springs on the Sinai Peninsula (Springs of Moses, Egypt). Bot. Lett. 165, 77–89. doi:10.1080/23818107.2017.1352535 37339054

[B55] Sans-Serramitjana E. Obreque M. Muñoz F. Zaror C. Mora M. Viñas M. . (2023). Antimicrobial activity of selenium nanoparticles (SeNPs) against potentially pathogenic oral microorganisms: a scoping review. Pharmaceutics 15, 2253. doi:10.3390/pharmaceutics15092253 37765222 PMC10537110

[B56] Schilrreff P. Alexiev U. (2022). Chronic inflammation in non-healing skin wounds and promising natural bioactive compounds treatment. Int. J. Mol. Sci. 23, 4928. doi:10.3390/ijms23094928 35563319 PMC9104327

[B57] Shah Z. Badshah S. L. Iqbal A. Shah Z. Emwas A. Jaremko M. . (2022). Investigation of important biochemical compounds from selected freshwater macroalgae and their role in agriculture. Chem. Biol. Technol. Agric. 9, 9. doi:10.1186/s40538-021-00273-0 38164791

[B58] Shahabi R. Anissian A. Javadmoosavi S. A. Nasirinezhad F. (2021). Protective and anti-inflammatory effect of selenium nanoparticles against bleomycin-induced pulmonary injury in male rats. Drug Chem. Toxicol. 44, 92–100. doi:10.1080/01480545.2018.1560466 31146593

[B59] Sharifi-Rad M. Elshafie H. S. Pohl P. (2024a). Green synthesis of silver nanoparticles (AgNPs) by Lallemantia royleana leaf extract: Their bio-pharmaceutical and catalytic properties. J. Photochem. Photobiol. A. Chem. 448, 115318. doi:10.1016/j.jphotochem.2023.115318 38826717

[B60] Sharifi-Rad M. Mohanta Y. K. Pohl P. Nayak D. Messaoudi M. (2024b). Facile phytosynthesis of gold nanoparticles using Nepeta bodeana Bunge: Evaluation of its therapeutics and potential catalytic activities. J. Photochem. Photobiol. A. Chem. 446, 115150. doi:10.1016/j.jphotochem.2023.115150 38826717

[B61] Sivasamy S. Ezhaveni S. Kim Y. G. Lee J. H. Lee J. (2020). Antibiofilm and antivirulence properties of indoles against Serratia marcescens. Front. Microbiol. 11, 584812. doi:10.3389/fmicb.2020.584812 33193228 PMC7662412

[B62] Snehalatha D. Rao B. D. (2017). Antibacterial activity of freshwater green algae, Chara vulgaris. Int. J. CRGG 10, 749–753. Available online at: https://sphinxsai.com/2017/ch_vol10_no6/2/(749-753)V10N6CT.pdf.

[B63] Swidan N. S. Hashem Y. A. Elkhatib W. F. Yassien M. A. (2022). Antibiofilm activity of green-synthesized silver nanoparticles against biofilm-associated enterococcal urinary pathogens. Sci. Rep. 12, 3869. doi:10.1038/s41598-022-07831-y 35264654 PMC8907169

[B64] Yadav G. Thakuria B. Madan M. Agwan V. Pandey A. (2017). Linezolid and vancomycin-resistant enterococci: a therapeutic problem. J. Clin. Diagn. Res. 11, GC07–GC11. doi:10.7860/JCDR/2017/27260.10474 28969155 PMC5620796

