# Supplementary material for: Chara vulgaris-mediated selenium nanoparticles: a novel approach to antioxidant, antibacterial, anti-inflammatory, and wound healing activities
Source: Front Cell Infect Microbiol. 2026 Jun 9;16:1752175. doi: 10.3389/fcimb.2026.1752175 (PMC13287021; doi:10.3389/fcimb.2026.1752175)
Supplement: Supplementary file 1 [file SupplementaryFile1.docx]

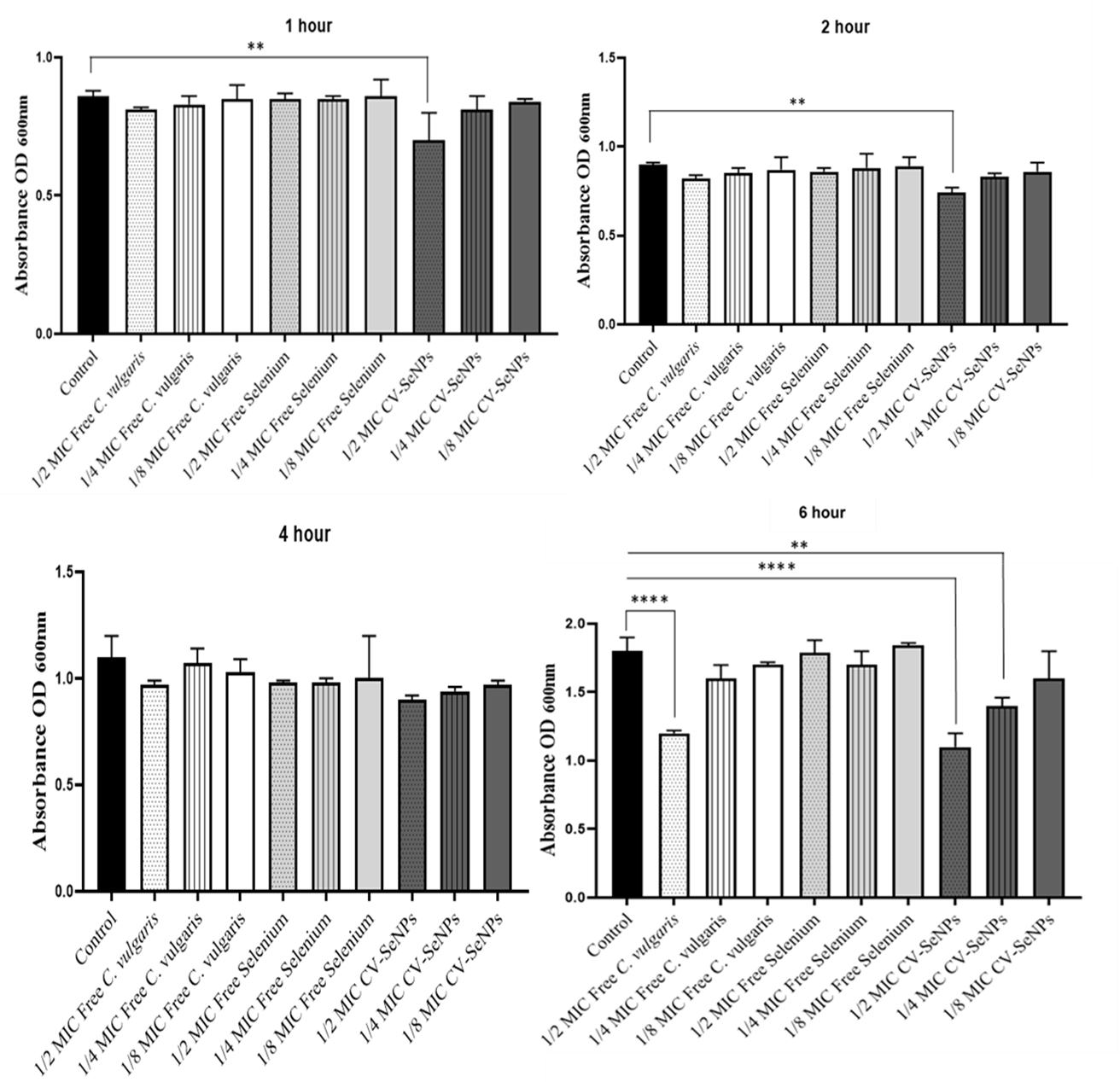


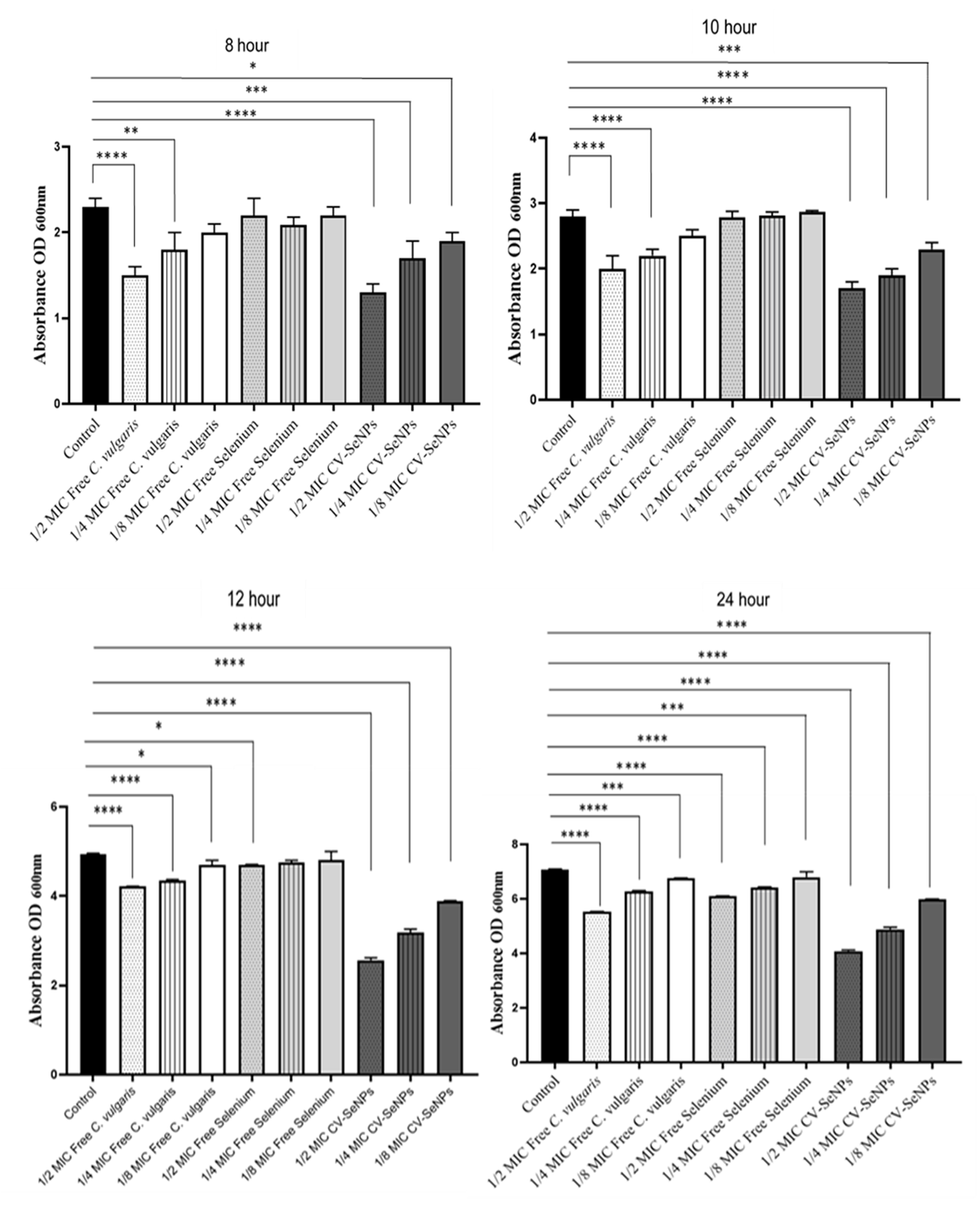


**Figure S1:** Statistical comparisons of growth curves between treated and untreated groups at different time intervals (One-way ANOVA, groups were compared using multiple comparisons with Tukey's). Error bars and asterisks (*) represent standard deviations and statistically significant differences (* *p < 0.05*, ** *p < 0.01*, *** *p < 0.001,* *** *p < 0.0001*), respectively.
